# PD80 Brazilian Supreme Court’s Decision On The Provision Of Non-Incorporated Medicines: Implications For The Health Technology Assessment Process

**DOI:** 10.1017/S0266462325102079

**Published:** 2025-12-29

**Authors:** Rafaella Maria Vasconcelos da Nóbrega, Laís da Silva Barbosa, Barbara Pozzi Ottavio, Thâmmara Lariane Henriques Tito, Marcela Medeiros de Freitas, Maria Stella Martins Silva D’agostini, Luciene Fontes Schluckebier Bonan

## Abstract

**Introduction:**

People frequently use litigation to access technologies in Brazil. From 2020 to 2023 there was a 38 percent increase in the lawsuits requesting drugs (55,600 to 76,700). The Ministry of Health’s expenditure on litigation reached USD428 million in 2023. This study analyzed the decision issued on September 2024 by the Brazilian Supreme Court (STF) to address litigation.

**Methods:**

The study reviewed the STF decision, identifying the requirements for judicial provision of medicines not incorporated into the public health system that are directly related to the health technology assessment process.

**Results:**

By a 10-to-one majority, the STF decided that medicines not incorporated into the public health system should not be judicially granted. Exceptions apply to medicines registered by the National Health Surveillance Agency and are based on several criteria: evidence of illegality in a decision by the National Committee for Health Technology Incorporation (CONITEC) or excessive delays in its analysis; lack of substitutes in the public health system; or scientific evidence of efficacy and safety. It was also concluded that CONITEC’s recommendation not to incorporate a drug should be considered, and that when a drug is judicially granted it could be submitted to CONITEC for assessment.

**Conclusions:**

The STF decision seeks to guarantee both the right to health and the financial sustainability of the public health system. By requiring robust scientific evidence for judicially granted medicines, it underscores CONITEC’s crucial role and the need for evidence-based decision-making at an individual level. Its determination also pushes for greater coordination between the Judiciary and Executive Branches of the government.

